# Resveratrol Enhances Antioxidant and Anti-Apoptotic Capacities in Chicken Primordial Germ Cells through m6A Methylation: A Preliminary Investigation

**DOI:** 10.3390/ani14152214

**Published:** 2024-07-30

**Authors:** Yanzhao Qiao, Gengsheng Xiao, Xiaohua Zhu, Jun Wen, Yonghui Bu, Xinheng Zhang, Jie Kong, Yinshan Bai, Qingmei Xie

**Affiliations:** 1 State Key Laboratory of Swine and Poultry Breeding Industry & Heyuan Branch, Guangdong Provincial Laboratory of Lingnan Modern Agricultural Science and Technology, College of Animal Science, South China Agricultural University, Guangzhou 510642, China; 2 College of Animal Science, South China Agricultural University, Guangzhou 510642, China; 3 Key Laboratory of Agricultural Animal Genomics and Molecular Breeding of Guangdong Province, College of Animal Science, South China Agricultural University, Guangzhou 510642, China; 4 Guangdong Academy of Agricultural Sciences, Guangzhou 510640, China; 5 Guangdong Provincial Key Laboratory of Animal Molecular Design and Precision Breeding, Foshan University, Foshan 528231, China

**Keywords:** primordial germ cells, resveratrol, antioxidant, apoptosis, m6A methylation

## Abstract

**Simple Summary:**

Avian primordial germ cells (PGCs) are vital for transgenic poultry research, germplasm preservation, and disease resistance breeding. However, these cells often encounter fragmentation and apoptosis during in vitro culture, limiting their broader application. This study demonstrates that adding resveratrol (RSV) to established PGC cultures enhances their antioxidant and anti-apoptotic capacities. m6A methylation analysis identified two candidate genes, *FAM129A* and *SFRP1*, closely associated with apoptosis, indicating potential for further investigation. Our findings reveal RSV’s protective effects and underlying mechanisms in chicken PGCs, offering new insight and a potential application for RSV as an optimal supplement in reproductive stem cell culture.

**Abstract:**

Avian primordial germ cells (PGCs) are essential in avian transgenic research, germplasm conservation, and disease resistance breeding. However, cultured PGCs are prone to fragmentation and apoptosis, regulated at transcriptional and translational levels, with N6-methyladenosine (m6A) being the most common mRNA modification. Resveratrol (RSV) is known for its antioxidant and anti-apoptotic properties, but its effects on PGCs and the underlying mechanisms are not well understood. This study shows that RSV supplementation in cultured PGCs improves cell morphology, significantly enhances total antioxidant capacity (*p* < 0.01), reduces malondialdehyde levels (*p* < 0.05), increases anti-apoptotic *BCL2* expression, and decreases *Caspase-9* expression (*p* < 0.05). Additionally, RSV upregulates the expression of m6A reader proteins *YTHDF1* and *YTHDF3* (*p* < 0.05). m6A methylation sequencing revealed changes in mRNA m6A levels after RSV treatment, identifying 6245 methylation sites, with 1223 unique to the control group and 798 unique to the RSV group. Combined analysis of m6A peaks and mRNA expression identified 65 mRNAs with significantly altered methylation and expression levels. Sixteen candidate genes were selected, and four were randomly chosen for RT-qPCR validation, showing results consistent with the transcriptome data. Notably, *FAM129A* and *SFRP1* are closely related to apoptosis, indicating potential research value. Overall, our study reveals the protective effects and potential mechanisms of RSV on chicken PGCs, providing new insight into its use as a supplement in reproductive stem cell culture.

## 1. Introduction

Primordial germ cells (PGCs) are progenitor cells that give rise to male and female gametes, ensuring the transgenerational transmission of genetic information. In poultry research, chicken PGCs play diverse roles. For instance, by introducing the human interferon β (hIFN-β) gene into recipient birds, PGCs have been shown to act as protein bioreactors [1]. Additionally, transgenic chickens expressing the EGFP gene on the Z chromosome, developed using PGCs, have advanced sex determination studies [2]. PGCs are also pivotal in creating disease-resistant chickens, such as the generation of ΔW38 haploid chickens, which show complete resistance to avian leukosis virus subgroup J (ALV-J) through W38 amino acid knockout [3]. Moreover, injecting cryopreserved PGCs into the embryonic vasculature to create chimeras and produce donor-derived offspring highlights PGCs’ potential in germplasm preservation [4]. However, the widespread application of PGCs depends on a stable and continuous in vitro supply, which is challenged by apoptosis and oxidative stress during long-term culture, leading to cell deformation and fragmentation [5,6,7].

Previous studies have identified oxidative stress as a potential pro-apoptotic factor that influences stem cell behavior by promoting apoptosis [8,9,10,11]. Under oxidative stress or prolonged passaging, stem cells experience senescence, loss of stemness, and functional impairment [12]. This effect is particularly detrimental to reproductive stem cells, potentially hindering the transmission of genetic information across generations. Therefore, employing exogenous antioxidants to mitigate and prevent oxidative stress is crucial.

Phenolic compounds are critical in mitigating oxidative stress and preventing cell death due to their optimal structural chemistry for free radical quenching [13,14]. Among these compounds, resveratrol (RSV), chemically known as trans-3,5,4′-trihydroxystilbene, is noteworthy. Predominantly found in red wine and various plants, including grapes, peanuts, and plums [15,16], RSV’s hydroxyl groups effectively scavenge hydroxyl and hydroperoxyl radicals, establishing RSV as a potent antioxidant [17,18,19,20,21,22]. In reproductive biology, RSV has been shown to prevent ovarian oxidative stress in rats with polycystic ovary syndrome (PCOS) by reducing malondialdehyde (MDA) levels and increasing superoxide dismutase (SOD) levels [23]. Additionally, RSV supplementation in rooster sperm cryopreservation media has been linked to improved sperm motility and enhanced total antioxidant capacity (T-AOC) [24]. Despite extensive research on RSV’s biological properties, its specific effects on chicken PGCs remain unexplored.

RNA methylation is a significant area of epigenetic research, encompassing processes such as transcription, mRNA translation, DNA damage response, heat shock response, sex determination, and gene expression regulation [25,26,27]. Among all types of RNA methylation, N6-methyladenosine (m6A) is the most common RNA modification, comprising about 60% of RNA methylations [28,29]. m6A plays a critical role in RNA metabolism and cellular function [30] and is closely linked to oxidative stress [31] and apoptosis [32]. Reports have indicated that abnormally elevated levels of m6A increase the apoptosis rate of human or mouse ovarian granulosa cells (GCs), impair ovarian function, and lead to premature ovarian insufficiency (POI) [33]. Therefore, maintaining m6A homeostasis may be crucial for monitoring oxidative stress and apoptosis. However, the relationship between the antioxidant and anti-apoptotic capacities of cultured chicken PGCs and their m6A status is unclear. This study aims to explore whether RSV affects the antioxidant capacity and apoptosis of chicken PGCs through m6A modification and to elucidate the underlying mechanisms.

## 2. Materials and Methods

### 2.1. Animals and Ethics

All animal procedures were approved by the South China Agricultural University Animal Protection and Utilization Committee and followed the Guiding Principles for the Protection and Utilization of Laboratory Animals.

### 2.2. RSV Treatment of Chicken PGCs

Primary PGCs were isolated from the gonads of 6–7-day-old White Leghorn chicken embryos, cultured in vitro, and characterized using molecular biology techniques for this study (see Supplementary Figures S1 and S2, and Table S1). The PGCs were uniformly seeded into 6-well plates containing a feeder layer of STO cells. After 48 h, the PGCs reached 70% confluence. The RSV treatment at this point was administered at a dosage determined based on previous research [34,35]; 1.5 μL of DMSO (Sigma-Aldrich, D2650, Irvine, UK) was added to each well in the control group (3 replicates), while 1.5 μL of RSV (50 μM, Sigma-Aldrich, R5010, dissolved in DMSO) was added to each well in the RSV group (3 replicates). After mixing, the cells were cultured for another 48 h. Subsequently, some PGCs from each well were collected for antioxidant capacity assays and RT-PCR analysis (methods detailed below), while the remaining PGCs were sent to Lianchuan Biological (Hangzhou, China) for m6A sequencing.

### 2.3. Determination of Antioxidant Capacity of PGCs

The total antioxidant capacity (T-AOC), malondialdehyde (MDA), superoxide dismutase (SOD), and glutathione peroxidase (GSH-Px) activities in chicken PGCs were measured using assay kits according to the China Nanjing Jiancheng Bioengineering Institute (Total Antioxidant Capacity Assay Kit, ABTS method, A015-2-1; Cell Malondialdehyde Assay Kit, colorimetric method, A003-4-1; Superoxide Dismutase Assay Kit, WST-1 method, A001-3-2; Glutathione Peroxidase Assay Kit, A005-1-1). At the same time, the protein concentration after homogenization of the cell samples was determined using the BCA Protein Quantification Kit (A045-3) of the same company.

### 2.4. RNA Extraction, RT-PCR, and qPCR Assays

Total RNA from PGCs in the RSV and control groups was extracted using Trizol (Beyotime, R0016) and then reverse-transcribed into cDNA with the StarScript III All-in-one RT Mix with gDNA Remover (GenStar, A234). Subsequently, the expression levels of several genes, including *BCL2*, *BAX*, *Caspase-3*, *Caspase-9*, *METTL3*, *METTL14*, *WTAP*, *FTO*, *ALKBH5*, *YTHDF1*, *YTHDF2*, *YTHDF3*, *SFRP1*, *FAM129A*, *SH3RF3*, and *KCNJ2*, were determined using the Real-Time PCR Easy^TM^-SYBR Green I Kit (Foregene, Chengdu, China). The total volume of the PCR reaction system was 20 μL, and the PCR cycling conditions were 95 °C for 30 s to denature the cDNA template, followed by 40 cycles at 95 °C for 10 s and annealing at melting temperature (Tm) for 30 s. Finally, the relative expression was calculated using the 2^−ΔΔCT^ method. Table S1 lists the primers used for the polymerase chain reaction.

### 2.5. Detection of m6A Methylation and Gene Expression Levels in PGCs

Total RNA was isolated and purified from cell samples using the Trizol method. Subsequently, RNA quality and purity were confirmed by agar electrophoresis. Magnetic beads containing polyadenylate were used for specific mRNA capture. Consequently, the RNA was fragmented using the Magnesium Ion Disruption Kit and premixed with an antibody having immunomagnetic beads m6A for IP protection. The obtained RNA samples were subsequently eluted and purified. MeRIP RNA was analyzed using RT-qPCR and matched with the corresponding input RNA. Then, the RNA-seq library was constructed. The DNA and RNA duplexes were converted into DNA duplexes with complementary ends to synthesize the IP product; both ends were added with base A, ligated to the ends with base T, and purified by magnetic bead screening. After digesting the double-stranded bodies, the PCR products underwent pre-denaturation at 95 °C for 3 min, denaturation at 98 °C for 15 s, 8 amplification cycles, annealing at 60 °C for 15 s, extension at 72 °C for 30 s, and held at 72 °C for 5 min to obtain approximately 300 bp-sized fragments. Finally, the sample was sequenced, and the data were analyzed using the Illumina NovaSe^TM^ 150 according to the PE6000 sequencing mode.

### 2.6. Statistical Analysis

GraphPad Prism version 9 (GraphPad Software, La Jolla, CA, USA) was used for statistical analysis. All results are presented as the mean ± standard deviation and plotted using SPSS 25.0 software (SPSS, Inc., Chicago, IL, USA). The independent samples *t*-test was employed with fixed effects treatment. *p* < 0.05 was considered significant.

## 3. Results

### 3.1. Effects of RSV on Antioxidant Capacity and Apoptotic Genes in Chicken PGCs

**Figure 1:**
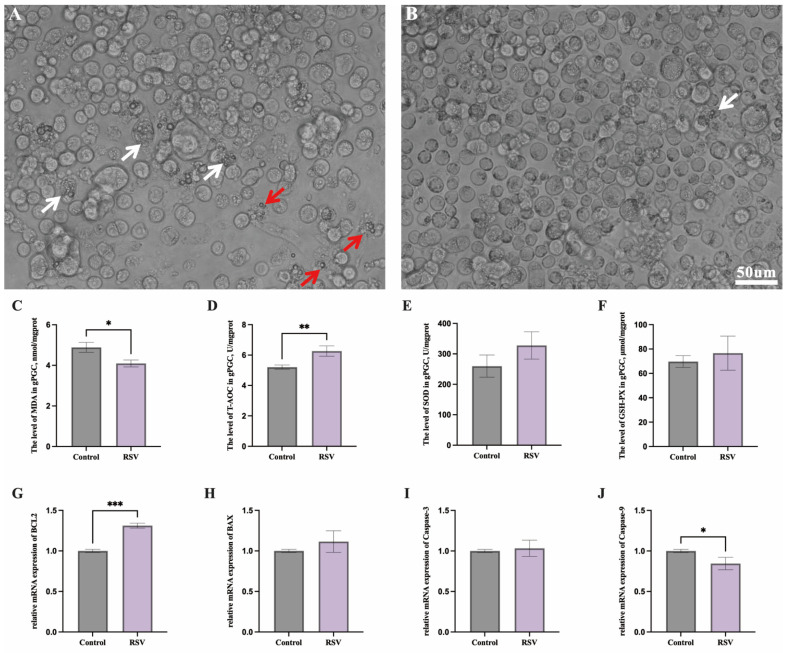
Effects of RSV on PGC morphology and the expression of antioxidant and apoptotic genes in chickens. Observation of PGCs in culture wells in control group (**A**) and RSV-treated group (**B**), with white and red arrows indicating deformed and fragmented PGCs, respectively. (**C**–**F**) Proof of changes in antioxidant parameters between RSV and control groups. (**G**–**J**) Quantitative analysis of apoptosis-related genes in RSV and control groups. * represents *p* < 0.05; ** *p* < 0.01; *** *p* < 0.001.

To study the effects of RSV on PGC growth status, the natural plant antioxidant RSV was added to established PGCs for 48 h of treatment. Under light microscopy, higher numbers of deformed (white arrows) and fragmented PGCs (red arrows) were observed in the control group (Figure 1A), while only a few deformed PGCs were found in the RSV-treated group (Figure 1B, white arrows). Subsequently, the changes in oxidative stress indexes and apoptosis gene expression in PGCs from both groups were investigated. Compared to the control group, the RSV group showed a significant decrease in MDA levels in PGCs (Figure 1C, *p* < 0.05) and a notable increase in T-AOC levels (Figure 1D, *p* < 0.01). Although the SOD and GSH-PX levels in the RSV group were higher numerically than those in the control group, the differences were not significant (Figure 1E,F, *p* > 0.05). The RT-qPCR data indicated that RSV significantly increased the expression of anti-apoptotic gene *BCL2* in chicken PGCs (Figure 1G, *p* < 0.001) and reduced the expression of pro-apoptotic gene *Caspase-9* (Figure 1J, *p* < 0.05) compared to the control group. However, the expression levels of *BAX* and *Caspase-3* did not differ significantly between the two groups (Figure 1H,I, *p* > 0.05).

### 3.2. Effects of RSV on Methylase, Demethylase, and Methylated Reading Proteins of PGCs

**Figure 2:**
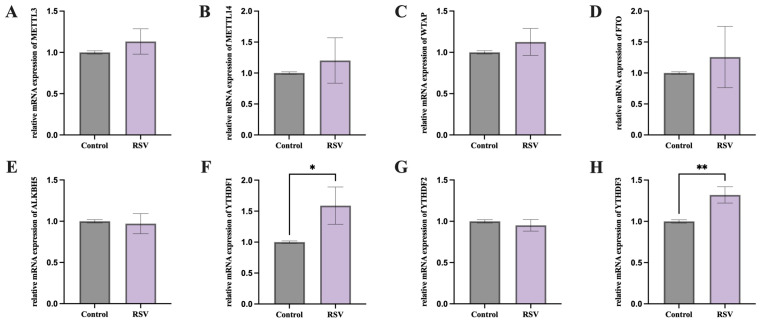
Effects of RSV on methylase, demethylase, and methylated reading protein RNA in PGCs. (**A**–**C**) Results of methylase, RT-qPCR. (**D**,**E**) Results of demethylase, RT-qPCR. (**F**–**H**) Results of methylated reading protein, RT-qPCR. * represents *p* < 0.05; ** *p* < 0.01.

We then explored the changes in gene expression of methylase, demethylase, and methylated reader proteins in the two groups of PGCs. Although there were no significant differences in gene expression levels of methylase and demethylase in the PGCs of the two groups, RSV significantly increased the expression of methylated reading proteins YTHDF1 (Figure 2F, *p* < 0.05) and YTHDF3 (Figure 2H, *p* < 0.01) in chicken PGCs compared to the control group.

### 3.3. m6A Methylation Peak Analysis of Chicken PGCs

**Figure 3:**
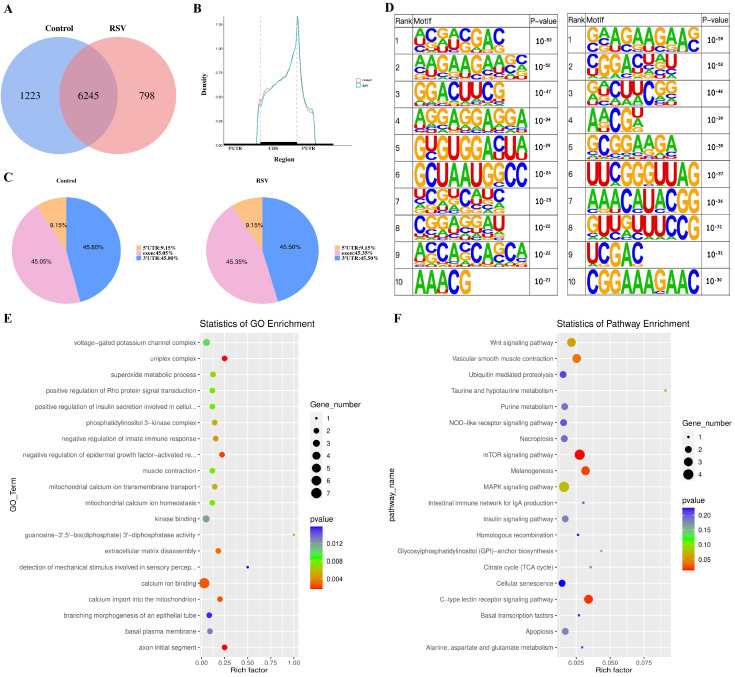
m6A methylation peak analysis. (**A**) The Wayne plots of m6A peak number in the control and RSV groups. (**B**) Distribution position of m6A sites on mRNA and (**C**) its percentage in samples from both groups. (**D**) Motif sequences containing m6A peaks with the top ten *p*-values in the control (**left**) and RSV groups (**right**). (**E**) GO and (**F**) KEGG analyses of differential peaks between the two groups.

PGCs were harvested from both the control and resveratrol (RSV)-treated groups, followed by methylation analysis using MeRIP-seq high-throughput sequencing to investigate the effect of RSV on the epigenetic regulation of chicken PGCs. The results showed that 1223 unique methylation sites were detected in the control group, while 798 unique methylation sites were detected in the RSV group. The total number of overlapping methylation sites between the two groups was 6245 (Figure 3A). Further results showed that the m6A peaks were mainly enriched in three parts: the 5′ untranslated region (5′ UTR), the 3′ untranslated region (3′ UTR), and the exon. Most of the m6A sites in the control and RSV groups were located in the 3′ UTR and coding region (CDs) (Figure 3B). Compared to the control group, the RSV group had a 0.3% reduction in sites in the 3′ UTR and a 0.3% increase in sites in the exon (Figure 3C). RNA methylation and demethylation changes are triggered by the attachment of multiple binding proteins to motifs, which are important modes for these proteins to recognize nucleic acid sequences, and are also the sites where methylation occurs. Figure 3D shows the motif sequence results for the control group and the RSV group. In this study, the highly frequent and similar motifs detected in the control and RSV groups may have been potential methylation sites in chicken PGCs involving “RRACH” motifs, including “GGACU.” These findings suggested that the m6A methylation pattern of chicken PGCs is similar to that of other species. Consequently, the findings from the m6A methylation analysis performed on these two sample groups could serve as a foundational basis for subsequent investigations. We then performed gene Ontology (GO) and Kyoto Encyclopedia of Genes and Genomes (KEGG) analyses of the observed differences in peaks between the control and RSV groups. Figure 3E shows the first 20 terms of the GO enrichment analysis of the differential peaks of the two groups of samples, which were mainly enriched in the negative regulation of epidermal growth factor activation, calcium ion binding, calcium ion entry into mitochondria, and other biological functions. Figure 3F shows the first 20 terms of the KEGG enrichment analysis of the peaks of the two groups of samples, which were significantly enriched in biological pathways such as the mTOR signaling pathway, the C-type lectin receptor signaling pathway, and vascular smooth muscle contraction.

### 3.4. Analysis of Transcriptional Gene Level

**Figure 4:**
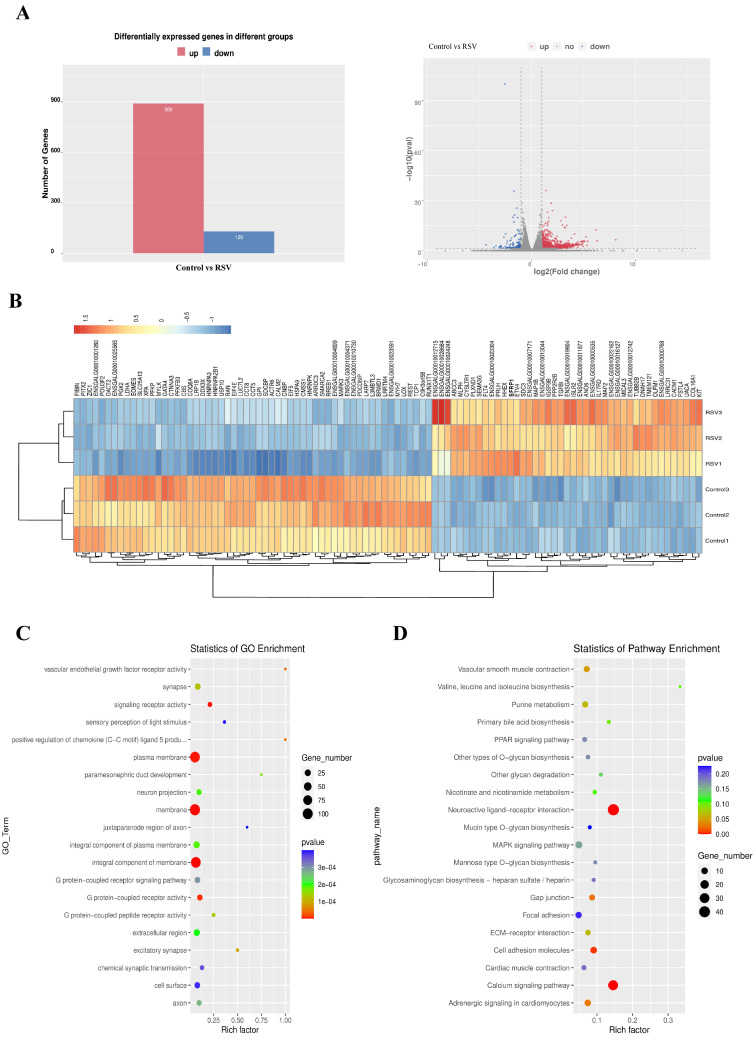
Analysis of transcriptional gene level. (**A**) The number of differentially differentiated genes upregulated and downregulated between the control and RSV groups (**left**), and the volcano map of differentially differentiated genes (**right**). (**B**) Cluster expression analysis of genes in the two groups of samples. (**C**) GO and (**D**) KEGG were used to analyze differential genes between the two groups.

Subsequent to the completion of transcriptome sequencing for cell samples from both the control and RSV-treated cohorts, a comprehensive analysis of differentially expressed genes was undertaken. The results showed that the number of upregulated genes (pink) was significantly higher than the number of downregulated genes (blue) between the two groups (Figure 4A). Subsequently, gene cluster analysis was performed for the two groups of differentially expressed genes (Figure 4B). In color, blue to red indicates low to high expression, respectively (log10(FPKM + 1)). Subsequently, the differentially expressed genes of the two groups were analyzed by GO and KEGG. Figure 4C shows the first 20 terms of the GO analysis, including vascular endothelial growth factor receptor activity, positive regulation of chemokine (c-c motif) ligand 5 products, and excitatory synapses. Figure 4D shows the top 20 terms analyzed by KEGG. These terms were mainly concentrated in neuroactive ligand-receptor interactions, gap junctions, cell adhesion molecules, and the calcium signaling, cardiomyocyte adrenergic signaling, and vascular smooth muscle contraction pathways.

### 3.5. Association Analysis of Differential Peaks with Differential Genes

**Figure 5:**
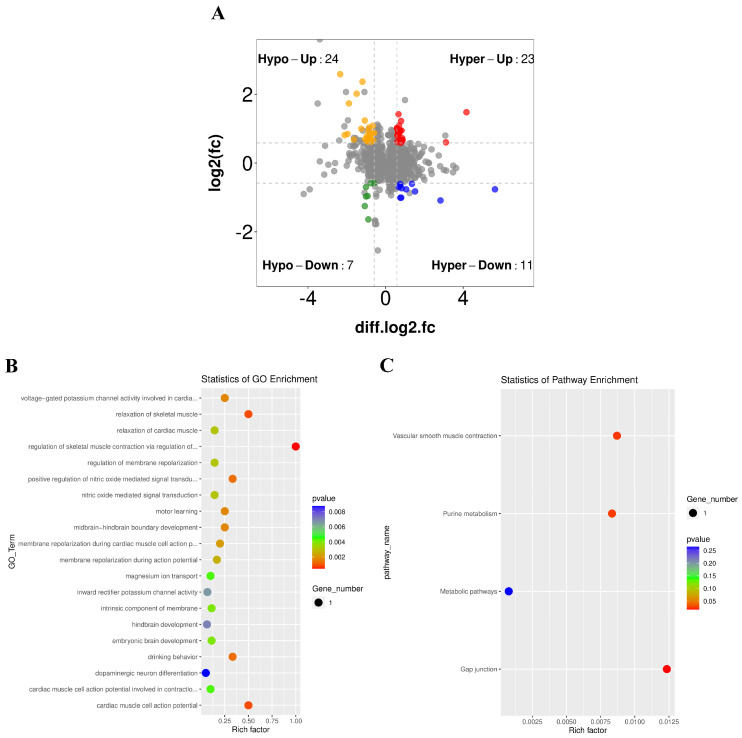
Association analysis of differential peaks and genes. (**A**) Quadruple image showing the number of genes with simultaneous changes in differential peaks and genes between the control and RSV groups: yellow represents the m6A peak and upregulated gene expression; red represents the downregulated m6A peak and upregulated gene expression; green represents m6A peak up-regulation, and gene expression downregulation; and blue represents m6A peak downregulation and gene expression downregulation. (**B**) GO analysis of differential peaks and gene associations between the two groups. (**C**) KEGG analysis of the association of differential peaks and genes between the two groups.

Analysis of the association between differential peaks and differential genes is crucial in genomics research, which helped to understand the gene expression and biological processes of chicken PGCs following RSV treatment. We conducted differential peak and differential gene association analysis on the data of the control group and RSV group, and set specific threshold screening conditions as *p* < 0.05 and Fc ≥ 1.5. The results of the four-image map showed that 65 genes with different peaks and different genes changed at the same time between the two groups. Among them, the yellow, red, green, and blue parts represent the distribution of genes with significant changes in both differential peaks and differential genes, respectively (Figure 5A). Subsequently, we performed GO and KEGG analyses on the results of the differential peak and differential gene association analysis in the control group and RSV group. GO analysis showed that the two groups were significantly enriched in regulating skeletal muscle contraction and relaxation through action potentials, positive regulation of cardiomyocyte action potentials, nitric oxide-mediated signal transduction, and voltage-gated potassium channel activity participating in repolarization of cardiomyocyte action potentials (Figure 5B). KEGG analysis showed significant enrichment in gap junction, vascular smooth muscle contraction, purine metabolism, and metabolic pathways in both groups (Figure 5C). In summary, m6A methylation modification under RSV treatment regulated the expression of several genes related to metabolism, growth, and differentiation in chicken PGCs.

### 3.6. Screening and Validation of Candidate Genes

After analyzing differential peaks and gene associations between the control and RSV groups, we identified 16 candidate genes with significant differences (*p* < 0.05, Fc ≥ 1.5). These genes exhibited notable changes in the association analysis, as shown in Table 1.

**Figure 6:**
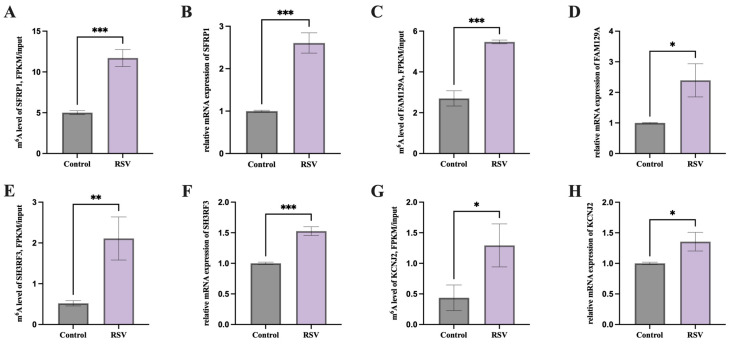
RT-qPCR validation of candidate genes. (**A**) m6A methylation level of *SFRP1* and (**B**) the mRNA expression level of the *SFRP1* gene, (**C**) m6A methylation level of *FAM129A* and (**D**) the mRNA expression level of the *FAM129A* gene, (**E**) m6A methylation level of *SH3RF3* and (**F**) the mRNA expression level of the *SH3RF3* gene, (**G**) m6A methylation level of *KCNJ2* and (**H**) the mRNA expression level of the *KCNJ2* gene, * represents *p* < 0.05; ** *p* < 0.01; *** *p* < 0.001.

We randomly selected 4 of the 16 candidate genes for MeRIP-PCR and RT-PCR validation. We found that the m6A methylation levels and mRNA expression of *SFRP1*, *FAM129A*, *SH3RF3*, and *KCNJ2* were significantly increased in the RSV group (Figure 6A–H, *p* < 0.05).

## 4. Discussion

The in vitro culture and expansion of avian PGCs are crucial for producing transgenic chickens and preserving avian genetic material [36]. However, apoptosis and oxidative stress in long-term PGC cultures have drawn attention to the antioxidant properties of resveratrol (RSV). This study explores the feasibility of using RSV to mitigate apoptosis and oxidative stress in avian PGCs and preliminarily investigates its mechanisms. The results indicate that RSV supplementation improves the morphology and integrity of PGCs and enhances their antioxidant and anti-apoptotic capacities. Furthermore, m6A methylation sequencing identified 16 candidate genes, among which *FAM129A* and *SFRP1* are closely related to the regulation of apoptosis. In conclusion, this study is the first to reveal that RSV may enhance the antioxidant and anti-apoptotic capabilities of chicken PGCs through the mechanism of m6A modification.

Oxidative stress and apoptosis are significant challenges in the in vitro culture of PGCs. During long-term culture, we observed morphological changes and fragmentation of PGCs (Figure 1A,B), indicating the need to optimize the culture system. Although our medium contained antioxidants such as β-mercaptoethanol (β-ME) and glutamine [37,38], oxidative stress and apoptosis persisted. The antioxidant effects of β-ME and glutamine may diminish over time and are insufficient for long-term culture. Therefore, we added resveratrol, which demonstrated significant antioxidant and anti-apoptotic effects (Figure 1C–J). Future studies could enhance the antioxidant capacity by incorporating additional antioxidants like vitamin C and N-acetylcysteine, which have been proven effective in other research [39,40]. Overall, optimizing culture conditions to maintain PGC stability is crucial for long-term studies, such as gene function research, disease model establishment, and poultry breeding technology development.

The intracellular REDOX system maintains organic balance and normal cell function [41], and T-AOC, as a comprehensive indicator of the cellular antioxidant system, reflects the overall antioxidant capacity of cells [42]. MDA is an important biomarker of oxidative stress, representing lipid peroxidation by free radicals, and can reflect oxidative damage in organisms [43,44]. Our study demonstrates that resveratrol enhances the total antioxidant capacity of PGCs and has a significant effect on inhibiting lipid peroxidation (Figure 1C,D). SOD is an important enzyme of the antioxidant defense system [45]. Previous studies have shown that both E- and Z-RSV play antioxidant roles in protecting bone marrow-derived mesenchymal stem cells from oxidative damage by promoting REDOX-related pathways [46]. In addition, natural RSV glycosides upregulate T-AOC and SOD in C. elegans, enhancing its resistance to oxidative stress [47]. However, our study found no significant changes in SOD and GSH-PX levels (Figure 1E,F). This may be because the activity of these enzymes was sufficient to maintain the basal intracellular REDOX balance. Additionally, the specific duration and dosage of RSV treatment in our experiments might have contributed to this observation. Different time points or higher doses of resveratrol could potentially yield different results. Apoptosis is a programmed death in which the number of apoptotic cells determines the degree of eventual cell damage [48,49]. *BCL2* inhibits apoptosis and promotes cell survival and resistance to damage, while *BAX* induces apoptosis [50]. RSV has been shown to effectively reduce neuronal cell apoptosis while upregulating *BCL2* and downregulating *Caspase-3* expression [51]. Further studies showed that RSV could promote the survival of H9c2 cells, increase the expression of *BCL2*, and decrease the expression of BAX [52], and similar results were obtained in this study (Figure 1G). Our previous studies have also shown that dietary supplementation with RSV can effectively improve the liver antioxidant capacity of yellow-plumed broilers and reduce the apoptosis and cytotoxicity in the porcine intestinal epithelial cell line (IPEC-J2) induced by deoxynivalenol [53,54]. In this study, our results showed that, after RSV treatment, the expression of apoptosis regulator *Caspase-9* gene in chicken PGCs was significantly reduced, while the expression of *Caspase-3* and *BAX* genes was not significantly changed. Previous reports have indicated that *Caspase-9* is a promoter of apoptosis, while *Caspase-3* is the ultimate executor of apoptosis [55], suggesting that RSV may play a regulatory role in the early stages of apoptosis program activation. PGCs are protected by inhibiting the initiation of apoptosis by reducing the expression of *Caspase-9*.

m6A methylation can perform different regulatory functions in cells under different environmental conditions [41,56]. m6A methylation is critical in oxidative stress-induced damage and apoptosis. In the cell REDOX reaction, m6A modification mainly affects the cell cycle, apoptosis, and senescence. During cobalt-induced oxidative stress, the expression of m6A demethylase *FTO* was inhibited, thereby regulating caspase activation, G1/S cell cycle arrest, and apoptosis in an m6A-dependent manner [57]. *METTL14* induced apoptosis of spinal cord neurons in patients with spinal cord injury by mediating m6A methylation of EEF1A2 [58]. In this study, following RSV treatment, only the mRNA levels of m6A reader proteins *YTHDF1* and *YTHDF3* were significantly elevated (Figure 2F,H). It is known that m6A reader proteins can recognize and bind to m6A-modified RNA to exert regulatory functions [59]. The upregulation of their expression suggests that RSV treatment may regulate oxidative stress and apoptosis in chicken PGCs by affecting m6A modifications and RNA metabolism. Subsequent MeRIP-seq high-throughput sequencing analysis confirmed that RSV treatment altered the overall m6A methylation levels in chicken PGCs. Although research on the impact of RSV on m6A methylation in chicken PGCs is limited, previous studies have shown that RSV alleviated high-fat diet-induced liver lipid homeostasis damage and reduced m6A methylation levels [60]. Our study found that RSV treatment significantly altered the function of genes associated with m6A methylation. Differentially methylated genes were primarily enriched in gap junctions, vascular smooth muscle contraction, purine metabolism, and metabolic pathways according to GO and KEGG analyses (Figure 3E,F).

m6A has been shown to influence several aspects of RNA biology, including splicing of mRNA precursors, regulation of RNA output from the nucleus, and regulation of mRNA translation processes [57]. Our results showed that 1223 unique methylation sites were detected in the control group and 798 unique methylation sites were detected in the RSV group, suggesting that resveratrol treatment does have a significant effect on methylation. Resveratrol, as a polyphenol compound, affects DNA methylation status through a variety of mechanisms, including affecting DNA methyltransferase activity and altering the availability of methyl donors. In addition to the direct effects of resveratrol, experimental conditions, cell state, and environmental factors may also affect methylation status. In the design of the experiment, we tried to control for these variables, but it is undeniable that these factors may still have some influence on the results. In this study, we found that PGCs underwent some degree of methylation modification when exposed to resveratrol. Notably, these modifications occurred primarily in the 3′ UTR region of the exon (Figure 3B,C). m6A modifications are retained after the cleavage of mature mRNA and affect mRNA expression and translation to a certain extent [32]. In this study, in RSV-treated PGCs, specific genes involved in regulating the metabolism, growth, and differentiation of chicken PGCs underwent m6A methylation modification, resulting in changes in gene expression that had an impact on cell phenotype. In this study, we set threshold conditions (*p* < 0.05 and Fc ≥ 1.5) to identify 16 candidate genes that exhibited significant differences between the two groups. We randomly selected 4 of the 16 candidate genes for further verification. These genes represented different biological processes and functions and showed significant changes in the m6A methylation sequencing data. Under our experimental conditions, the expression levels of these genes were high enough for MeRIP-PCR and RT-PCR validation. Validation experiments showed that the m6A methylation levels of the four genes were consistent with the sequencing data, which supported the reliability of the sequencing results. Among the 16 candidate genes, it has been reported that *SH3RF3* is able to maintain the stem cell-like characteristics of cancer cells [61] and also regulates the proliferation, migration, and invasion of thyroid papillary cancer cells [62]. *KCNJ2* regulates cell growth cycle and drug resistance [63,64]. Downregulation of *KCNJ2* inhibited the metastasis of OS cells, while elevation of *KCNJ2* had the opposite effect [65]. *FAM129A* is a downstream molecule of activating transcription factor 4 (ATF4) and is known to inhibit apoptosis and promote tumor cell migration and proliferation [66]. Additionally, it has been reported to mediate apoptosis through the regulation of P53 [67]. Another candidate gene, *SFRP1*, is a regulatory factor in the Wnt/beta-catenin pathway involved in apoptosis, cell differentiation, and signal transduction [68,69]. *SFRP1* protects H9c2 cardiomyoblasts from adriamycin-induced apoptosis by inhibiting the Wnt/PCP-JNK pathway [70]. Increased expression of *SFRP1* has also been shown to inhibit apoptosis in fibroblast-related cells [71]. Our study found that the addition of RSV reduced the apoptosis level of PGCs and increased the expression of candidate genes *FAM129A* and *SFRP1*. This suggests that RSV may ultimately inhibit cell apoptosis by regulating the expression of *FAM129A* and *SFRP1*.

The limitations of this study include that it was conducted only on PGCs from White Leghorn chickens, and therefore, we cannot ascertain whether RSV would produce similar results when applied to PGCs derived from other breeds, necessitating further investigation. Additionally, the study did not delve deeply into the specific mechanisms by which RSV regulates the candidate genes, requiring further exploration of these mechanisms. Finally, while the use of 50 μM RSV as a supplement in the PGC culture system may have met the experimental needs of this study, it is necessary to determine the optimal concentration range for RSV as a potential supplement in PGCs culture media in future research.

## 5. Conclusions

This study is the first to demonstrate that RSV can improve the morphology of chicken PGCs, enhance their antioxidant and anti-apoptotic capabilities, and upregulate the expression of methylation reader proteins *YTHDF1* and *YTHDF3*. m6A methylation sequencing and analysis further identified candidate genes *FAM129A* and *SFRP1*, which are closely related to apoptosis and possess potential research value. These findings highlight the positive impact of RSV on the culture of chicken PGCs, providing new insight and potential applications for RSV in reproductive stem cell culture.

## Figures and Tables

**Table 1:** Information of the 16 candidate genes selected.

Gene Name	Seqname	m6A_Regulation	Gene_Regulation
*ANO6*	chr1	down	up
*SH3RF3*	chr1	down	up
*GUCY1A2*	chr1	up	down
*PRSS23*	chr1	down	up
*MAP2*	chr7	up	up
*EN1*	chr7	down	down
*FAM129A*	chr8	up	up
*FOXB1*	chr10	down	down
*CEP89*	chr11	up	down
*IL17RD*	chr12	up	up
*ENSGALG00010001642*	chr16	down	up
*IER5L*	chr17	down	up
*KCNJ2*	chr18	up	up
*CDR2L*	chr18	down	up
*SFRP1*	chr22	up	up
*ENSGALG00010005920*	chr31	down	up

## Data Availability

The original contributions presented in the study are included in the article. Further inquiries can be directed to the corresponding authors. The datasets used during the present study are available in the GEO (https://www.ncbi.nlm.nih.gov/geo/) database, accessed on 5 January 2024, accession number GSE48556.

## Supplementary Materials

The following supporting information can be downloaded at: https://www.mdpi.com/article/10.3390/ani14152214/s1, Figure S1. Related to RSV Treatment of Chicken PGCs; Figure S2. Related to RSV Treatment of Chicken PGCs; Table S1. PCR primers.
