# Supplementary material for: Resveratrol Enhances Antioxidant and Anti-Apoptotic Capacities in Chicken Primordial Germ Cells through m6A Methylation: A Preliminary Investigation
Source: Animals (Basel). 2024 Jul 30;14(15):2214. doi: 10.3390/ani14152214 (PMC11311097; doi:10.3390/ani14152214)
Supplement: Supplementary file 1 [file animals-14-02214-s001.zip › animals-3123129-supplementary.pdf]

Supplemental Information

Resveratrol Enhances Antioxidant and Anti-apoptotic Capacities in Chicken Primordial Germ Cells Through m6A Methylation: A Preliminary Investigation

Yanzhao Qiao, Gengsheng Xiao, Xiaohua Zhu, Jun Wen, Yonghui Bu, Xinheng Zhang, Jie Kong, Yinshan Bai and Qingmei Xie

Supplemental Figures

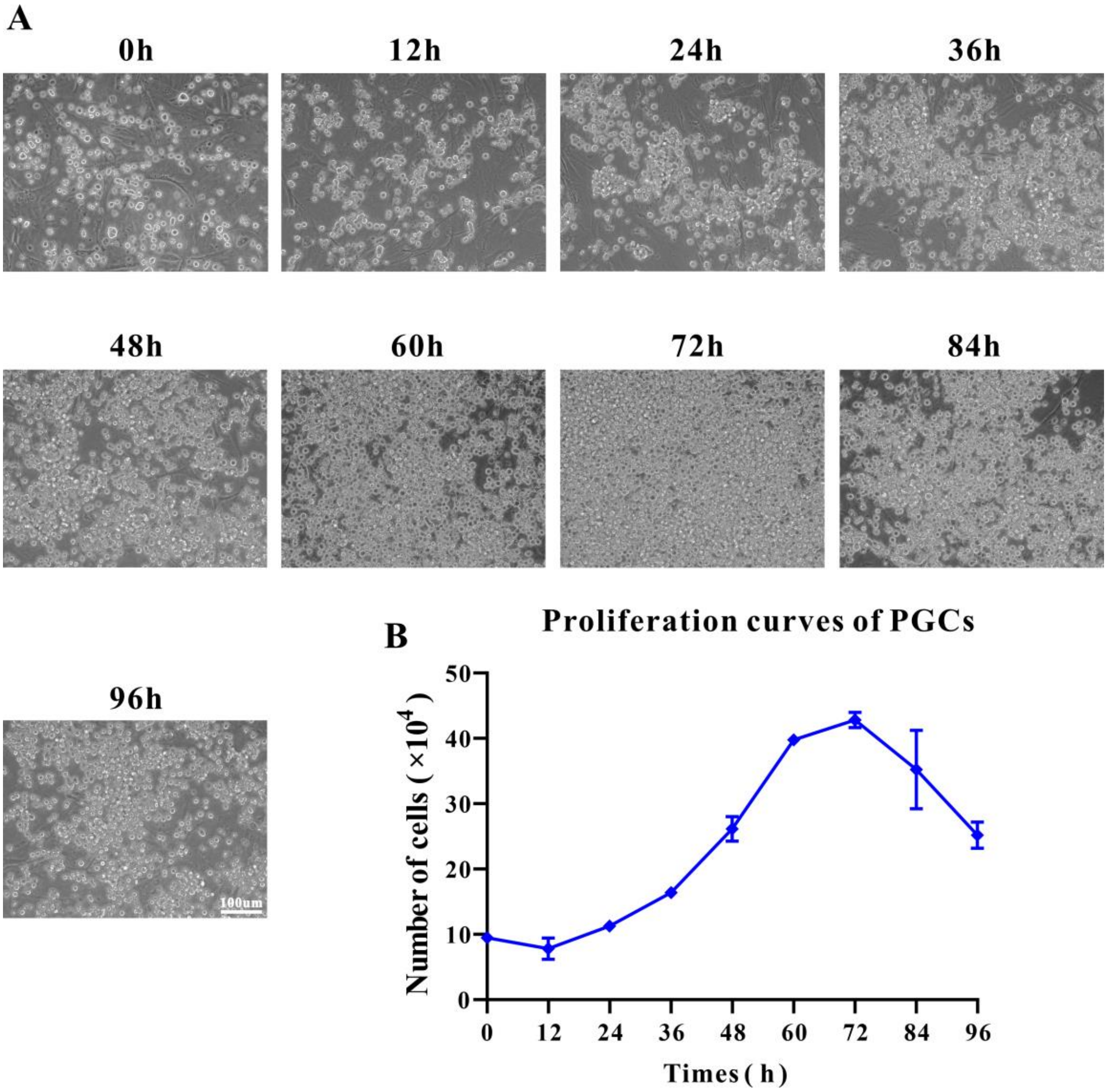

Figure S1. Related to RSV Treatment of Chicken PGCs. *In vitro* proliferation of chicken PGCs. **(A)** PGCs were isolated from the gonads of White Leghorn chicken embryos aged 6-7 days and cultured for a period of three weeks until reaching a cell count of $1×10^6$, indicating successful establishment of the PGC line. The established PGC lines were subsequently cultured on STO feeder layers for four days, with cell growth monitored at 12-hour intervals. **(B)** Corresponds to that shown in Figure A. The *in vitro* proliferation of the established PGC lines exhibited an "S" shaped growth curve, with a calculated cell doubling time of 48 hours.

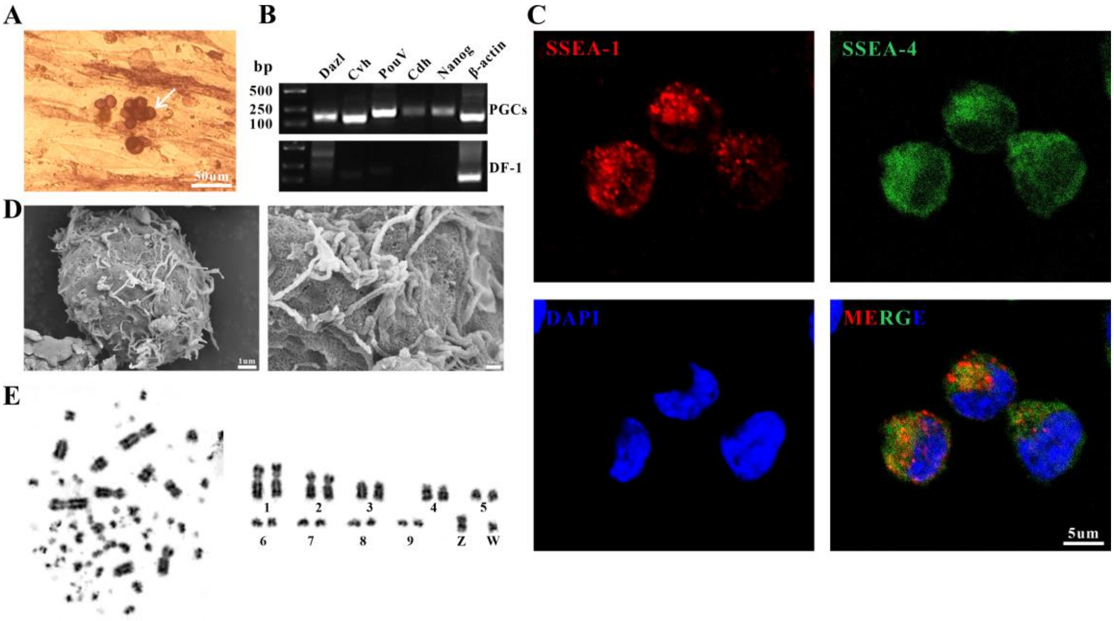

Figure S2. Related to RSV Treatment of Chicken PGCs. Molecular characterization of chicken PGCs. **(A)** Chicken PGCs cultured *in vitro* showed positive alkaline phosphatase staining (white arrow). **(B)** RT-PCR analysis detected the expression of germ cell-specific genes *Dazl*, *Cvh*, *PouV*, *Cdh*, and *Nanog* in chicken PGCs, whereas control DF-1 cells expressed only *β-actin*. **(C)** Immunofluorescence confocal microscopy revealed the surface expression of SSEA-1 (red) and SSEA-4 (green) in chicken PGCs. **(D)** Scanning electron microscopy showed that chicken PGCs were spherical, with surfaces covered by microvilli of varying lengths. **(E)** Karyotype analysis indicated that White Leghorn chicken PGCs had 39 pairs of chromosomes, including one pair of sex chromosomes (**Z and W**) and nine easily distinguishable pairs of autosomes.

Supplemental Experimental Procedures

Isolation and In Vitro Culture of PGCs

Specific pathogen-free (SPF) fertilized eggs of the White Leghorn chicken breed were provided by Xinxing Dahua Nong Poultry Eggs Co. and incubated for 6–7 days at 37.8 °C and 65% relative humidity. PGC isolation and culture followed previous reports with optimization [1,2]. A total of 35 chicken embryos aged between 6 and 7 days and exhibiting healthy developmental characteristics were selected. The selection was based on the assessment of their vascularization and overall embryonic development using an egg illuminator. Subsequently, the gonadal tissues of the embryos were carefully dissected using fine forceps and ophthalmic scissors. The tissue was then digested with 0.05% trypsin/EDTA for 50 min to isolate single cell. The resulting cell suspensions were mixed and seeded into a 6-well cell culture plate (Corning, NY), and the somatic cells were allowed to adhere to the bottom for 5 h. Suspended cells or cell clones exhibiting prominent refractive edges under the microscopic observation were identified as PGCs. The PGCs cells were passed to a 24-well culture plate and grown in KO-DMEM (Invitrogen, Carlsbad, CA) with 35% BRL conditioned medium [3], 7.5% fetal bovine serum (HyClone, Logan, UT) 2.5% chicken serum (Gibco, Carlsbad, California), 2 mM of glutamine (Invitrogen, Carlsbad, California), 1 mM of sodium pyruvate (Gibco, Carlsbad,

California), 1x of non-essential amino acids (Gibco, Carlsbad, California), 1x of nucleotides (Millipore, Bedford, Massachusetts), 1x of Antibiotic-Antimycotic (Gibco, Carlsbad, California), and 0.1 mM of β-mercaptoethanol (Gibco, Carlsbad, California), 6 ng/mL of stem cell factor (R&D System, Minneapolis, Minnesota), and 4 ng/mL of basic fibroblast growth factor (R&D System, Minneapolis, Minnesota). Feeder cells were seeded into the culture wells at a density of 2.5×10^4 cells/cm^2 1-2 days prior. These feeder cells, derived from γ-irradiated STO cell lines, secrete specific growth factors to promote the proliferation of PGCs.The PGCs were co-cultured with feeder layers that secrete growth factors specifically designed to facilitate their proliferation. All cell cultures were maintained in an incubator (Thermo, Wilmington, MA) set at 37°C with a 5% CO_2 concentration and 95% relative humidity.

Subculturing of primary PGCs cultures was commenced when the suspension-cultured PGCs reached approximately 50% confluence, typically 4-5 days post-inoculation. The suspended PGCs were collected in centrifuge tubes and centrifuged at 300 x g for 5 min. Half of the medium was discarded, and the other half was replaced with fresh medium to resuspend the cell pellet. The resulting suspension was seeded into a 12-well plate containing the feeder layer at 10,000 cells/mL density. When the confluence reached 80%, the PGC cell lines were subcultured at three-day intervals.For cryopreservation, PGCs were harvested upon reaching 90% confluence, resuspended in PGC medium supplemented with 10% DMSO and aliquoted in 1 mL volumes per cryovial. The prepared cryopreserved tubes with PGCs were placed in a cooling box (Corning, CoolCell) , designed for alcohol-free freezing at a controlled rate of -1°C/minute within a -80°C refrigerator. The next day, the tubes were transferred to a liquid nitrogen tank, maintaining a temperature of -196°C for long-term storage.

PGC Growth Curve Determination

The established cell lines were collected to form a uniform suspension and then seeded into 12-well plates coated with the STO feeder-layer. The initial seeding density of PGCs in each well was about 9.0×10^4/ml, recorded as time 0h. Subsequently, the PGC number from three randomized culture wells was recorded at time 0, 12, 24, 36, 48, 60, 72, 84 and 96 h.-Simultaneously, cell morphology, confluence, and fragmentation were documented using microscopic imaging. Subsequently, the acquired data were processed using GraphPad Prism9 (GraphPad Software, La Jolla, CA). Growth curves were plotted by time (h) as the horizontal axis and the number of cells ($\times 10^4$) at respective time points as the vertical axis. Finally, the mathematical formula available on the website (https://www.omnicalculator.com/biology/cell-doubling-time) was applied to calculate the doubling time of PGCs.

PGC Alkaline Phosphatase Staining

The BCIP/NBT Alkaline Phosphatase Color Development Kit (Beyotime, C3206) was used to identify *in vitro* cultured PGCs. Briefly, PGC attached to the feeder layer in 12-well plates was fixed with 4% paraformaldehyde (Leagene Biotechnology, Inc. DF0135) for 30 min. Subsequently, they were washed thrice with 1 mL of phosphate-buffered saline (PBS) buffer (Gibco) for 1 min each time. After washing, 1 mL of BCIP/NBT staining solution was added to each culture well, covering the cells adequately. The cells were then incubated at room temperature for 1.5 h protecting from light. After color development to the expected shade, the staining solution was aspirated and discarded, and the cells were washed twice with PBS. Images were taken using an inverted microscope (Olympus, CKX41).

RNA Extraction, RT-PCR Assays

The total RNA of DF-1 and PGCs was extracted with Trizol (Beyotime, R0016) and reverse-transcribed into cDNA with StarScript III All-in-one RT Mix with gDNA Remover (GenStar, A234). The PCR amplification of PGC related genes including *Dazl* 、*Cvh*、*PouV*、*Cdh*、*Nanog* and *β-actin* were performed with 2× Rapid Taq Master Mix (Vazyme, P222) under the following conditions: the PCR products underwent pre-denaturation at 95 °C for 3 min, denaturation at 95 °C for 30 s, annealing at 60 °C for 30 s, extension at 72 °C for 30 s, for 35 cycles, and a final extension at 72 °C for 7 min. Subsequently, the PCR products were subjected to agarose gel electrophoresis and visualized using an imaging system (Tanon, Shanghai). Table S1 lists the primers used for polymerase chain reaction.

Immunofluorescence Detection of PGCs

Coverslips treated with a poly-L-lysine solution from Sigma (P4832) were positioned into the bottom of 6-well plates in advance. Then PGCs were collected and prepared into single cell suspension with PBS-1% BSA solution and smeared. 4% paraformaldehyde from Leagene Biotechnology (DF0135) was added to fix 6-well plates at room temperature for 30 min. After washing, the cells were permeabilized using 0.2% Triton X-100 solution (Beyotime, P0096) and treated with a blocking solution (3% BSA solution) for 1 h. Next, the primary antibody working solution (1:15, SSEA-1/4, DSHB) was added and incubated at 4 °C for overnight. The plates were washed with PBS and incubated with the secondary antibody working solution (1:100, donkey anti-mouse-488, Abcam) at room temperature for 2 h. After rinsing, the PGCs were treated with a DAPI working solution (1:10,000, sigma) for 3 min, washed with PBS, and left to air-dry at room temperature with coverslips. Anti-fluorescence quenching sealer was added, and the samples were photographed using an ultra-high resolution laser confocal microscope (TCS SP8 STED 3X, Germany, LEICA).

Scanning Electron Microscopy Analysis of PGCs

In vitro cultured PGCs were collected, and the supernatant was discarded after centrifugation. The resulting cell pellet were fixed using 2.5% glutaraldehyde (Solarbio, P1126) for 6h at 4°C, followed by washing with PBS. The cells were fixed with 1% osmium tetroxide for 2 h and washed with PBS as previous described by **[4].** The samples underwent a sequential dehydration process using medical-grade alcohol at progressively increasing concentrations (50%, 70%, 80%, 90%) with each stage lasting 10 minutes, followed by two successive treatments with anhydrous ethanol, each lasting 10 minutes. Finally, the cells were dehydrated by immersing in hexamethyl disilazane (HMDS) for 2 hours. This process results in preserving the morphological integrity of the cells while enabling complete dehydration. The extreme-resolution analytical field emission SEM (model Tescan Mira 3XH) was used to image the samples.

Karyotyping of PGC Chromosomes

PGCs were pre-treated with medium containing 1% colchicine for 2 h to arrest mitosis and maintain chromosomes in mid-mitosis. Subsequently, the collected cells were washed of supernatant and resuspended in 5 mL of hypotonic solution at room temperature for 30 min. After centrifugation at 300 x g for 5 min, the cells were washed and resuspended in 5 mL of fixative solution (methanol: glacial acetic acid in a 3:1 ratio), fixed for 10 min at room temperature, followed by another centrifugation at 200 x g for 5 min and a final fixation. After

discarding a portion of the supernatant, the remaining fixative was used to resuspend the cells to create a homogeneous suspension. Subsequently, the cell suspension was aspirated and dropped onto a slide that had been pre-cooled at 4 °C. After drying the slide adequately, it was submerged in a 10% Gimsa staining solution for 30 min. Finally, the excess stain was washed off, the slides were dried and imaged under an orthogonal light microscope (OLYMPUS, CX33), and the chromosome arrangement was referred to in the previous paper [5,6].

TABLE S1 | PCR primers.

Gene	Primer sequence（5→3）	Gene bank	Tm（℃）
Dazl	F: TCCCAGAGCCCACACAGATG<br>R: AAGTGATGCGCCCTCCTCTC	XM_046910203.1	60.2
Cvh	F: CCTTGCAGCCTTTCTTTGTC<br>R: GCCTCTTGATGCTACCGAAG	XM_046934712.1	56.0
PouV	F: GTTGTCCGGGTCTGGTTCT<br>R: GTGGAAAGGTGGCATGTAGAC	NM_001309372.2	57.5
Cdh	F: CAACCGGACCAATAAGATGG<br>R: TGAGCGTGTCCTCATACAGG	XM_040682826.2	56.8
Nanog	F: CAGCAGACCTCTCCTTGACC<br>R: TTCCTTGTCCCACTCTCACC	XM_046906232.1	58.1
β-actin	F: CATTGTCCACCGCAAATGCT<br>R: AAGCCATGCCAATCTCGTCT	NM_205518.2	57.2
BAX	F: CAGCTCAGTGCAGCCGTC<br>R: ACAGAGAGCAAAGCTGCCAT	XM_040693909.2	59.4
BCL2	F: GATGACCGAGTACCTGAACC<br>R: CAGGAGAAATCGAACAAAGGC	XM_046910476.1	56.0

Caspase-3	F: TGGTGGAGGTGGAGGAGC<br>R: CATCTTCCCCTGAGCGTGG	XM_046915477.1	58.0
Caspase-9	F: TCCCGGGCTGTTTCAACTT<br>R: CCTCATCTTGCAGCTTGTGC	XM_046931415.1	59.0
SFRP1	F: GAAGAAGTTGGTGCTGCTCCTG<br>R: GTAGATGGCTGTCAGAAGGTGTTG	NM_204553.5	58.0
FAM129A	F: GGAGCTCCTTCCCAACCTTC<br>R: CGAATGGTGCCCTCAAGAGT	NM_001012596.3	59.0
SH3RF3	F: TCAGCTTCCCTACGGCAAAG<br>R: GCTCCCCGTGATACCAGTTC	XM_015277864.3	58.5
KCNJ2	F: AGGATCACGTCAGAAGGGGA<br>R: GGGAGACCAGGAATGCGG	NM_205370.2	57.5
METTL3	F: CTGACCGACGACGAGATGAG<br>R: CGTTCGTAGCCCCAAAGGTT	XM_040655036.2	59.0
METTL14	F: CTGCACCAAATGCAAAACGC<br>R: TTCTGGGGTCTATGTCCCGT	NM_001031148.2	58.0
WTAP	F: TCTCCCAAAGAAGGTTCGCC<br>R: GGTCTGTGTACTTGCCCTCC	XM_025148833.3	58.0
FTO	F: GAGCCTGGTCCTCCAAGAAG<br>R: GAAGGGATGGCATTCTGGCT	XM_040707054.2	59.0
ALKBH5	F: TCCCACTGGTTTGTGTTTGCC<br>R: CTGCCAACACTTGGGATCAAA	XM_004945197.5	59.0
YTHDF1	F: CCAACACATCCCGGAGTCAA<br>R: TCATCTCTGCTGAAGGGGGA	NM_001396237.1	58.0
YTHDF2	F: CCTGTCACTGTGGCACTCTT<br>R: GTTGCATCAGCAAACCAGCA	XM_004949271.5	58.0
YTHDF3	F: GCTCTCCCTGGTTTTTCCGAC<br>R: TGAGAAAACCTCGAAGCCCTG	NM_001006391.2	60.0

Supplemental References

1. Song, H.; Liu, D.; Wang, L.; Liu, K.; Chen, C.; Wang, L.; Ren, Y.; Ju, B.; Zhong, F.; Jiang, X. Methyltransferase like 7B is a potential therapeutic target for reversing EGFR-TKIs resistance in lung adenocarcinoma. *Molecular cancer* **2022**, *21*, 1-20.
2. Van de Lavoir, M.C.; Diamond, J.H.; Leighton, P.A.; Mather-Love, C.; Heyer, B.S., Bradshaw; Etches, R.J. Germline transmission of genetically modified primordial germ cells. *Nature* **2006**, *441*, 766-769.
3. Collarini, E.J.; Leighton, P.A.; Van de Lavoir, M.-C. Production of transgenic chickens using cultured primordial germ cells and gonocytes. *Microinjection: Methods and Protocols* **2019**, 403-430.
4. Choi, J.W.; Kim, S.; Kim, T.M.; Kim, Y.M.; Seo, H.W.; Park, T.S.; Jeong, J.-W.; Song, G.; Han, J.Y. Basic fibroblast growth factor activates MEK/ERK cell signaling pathway and stimulates the proliferation of chicken primordial germ cells. *PloS one* **2010**, *5*, e12968.
5. Ladjali-Mohammedi, K.; Bitgood, J.; Tixier-Boichard, M.; Ponce de Leon, F. International system for standardized avian karyotypes (ISSAK): standardized banded karyotypes of the domestic fowl (Gallus domesticus). *Cytogenetic and Genome Research* **1999**, *86*, 271-276.
6. Guttenbach, M.; Nanda, I.; Feichtinger, W.; Masabanda, J.S.; Griffin, D.K.; Schmid, M. Comparative chromosome painting of chicken autosomal paints 1–9 in nine different bird species. *Cytogenetic and Genome Research* **2003**, *103*, 173-184.
